# Supplementary material for: Environmental enrichment increases transcriptional and epigenetic differentiation between mouse dorsal and ventral dentate gyrus
Source: Nat Commun. 2018 Jan 19;9:298. doi: 10.1038/s41467-017-02748-x (PMC5775256; doi:10.1038/s41467-017-02748-x)
Supplement: Supplementary file 1 — Supplementary Information [file 41467_2017_2748_MOESM1_ESM.pdf]

Supplementary Information for

Environmental enrichment increases transcriptional and epigenetic differentiation between mouse dorsal and ventral dentate gyrus

Tie-Yuan Zhang[1,2,3,†,*], Christopher L Keown[4,†], Xianglan Wen[1,2,3], Junhao Li[4], Dulcie A. Vousden[5], Christoph Anacker[1,2,3], Urvashi Bhattacharyya[4], Richard Ryan[1,2,3], Josie Diorio[1,2,3], Nicholas O'Toole[1,2,3], Jason P. Lerch[,5], Eran A. Mukamel[4,*], Michael J. Meaney[1,2,3,6]

*Correspondence to: Tie-Yuan Zhang (tieyuan.zhang@mcgill.ca) and Eran A. Mukamel (emukamel@ucsd.edu).

†These authors contributed equally

This PDF file includes:

Supplementary Figures 1-5

Other Supplementary Materials for this manuscript include:

Supplementary Data 1

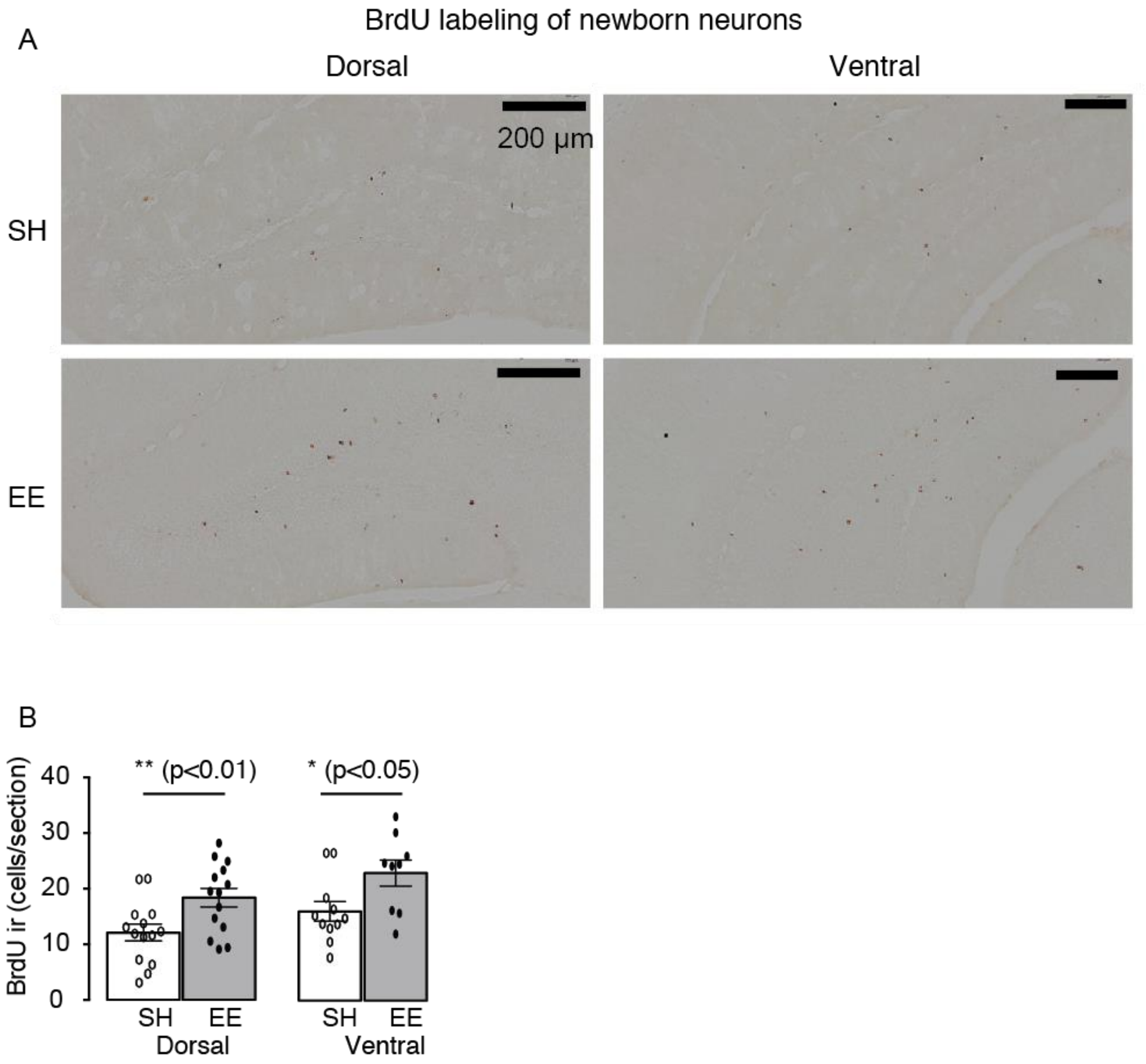

Supplementary Fig. 1 : Increased adult neurogenesis in dorsal and ventral DG following environmental enrichment. (A) BrdU immunoreactive cells in coronal section of dorsal and ventral dentate gyrus of mice. (B) Mean ± SEM BrdU-positive cells in dorsal and ventral dentate gyrus of mice (n = 9-14 per group, $F(1,44) = 13.33$, $p = 0.0007$).

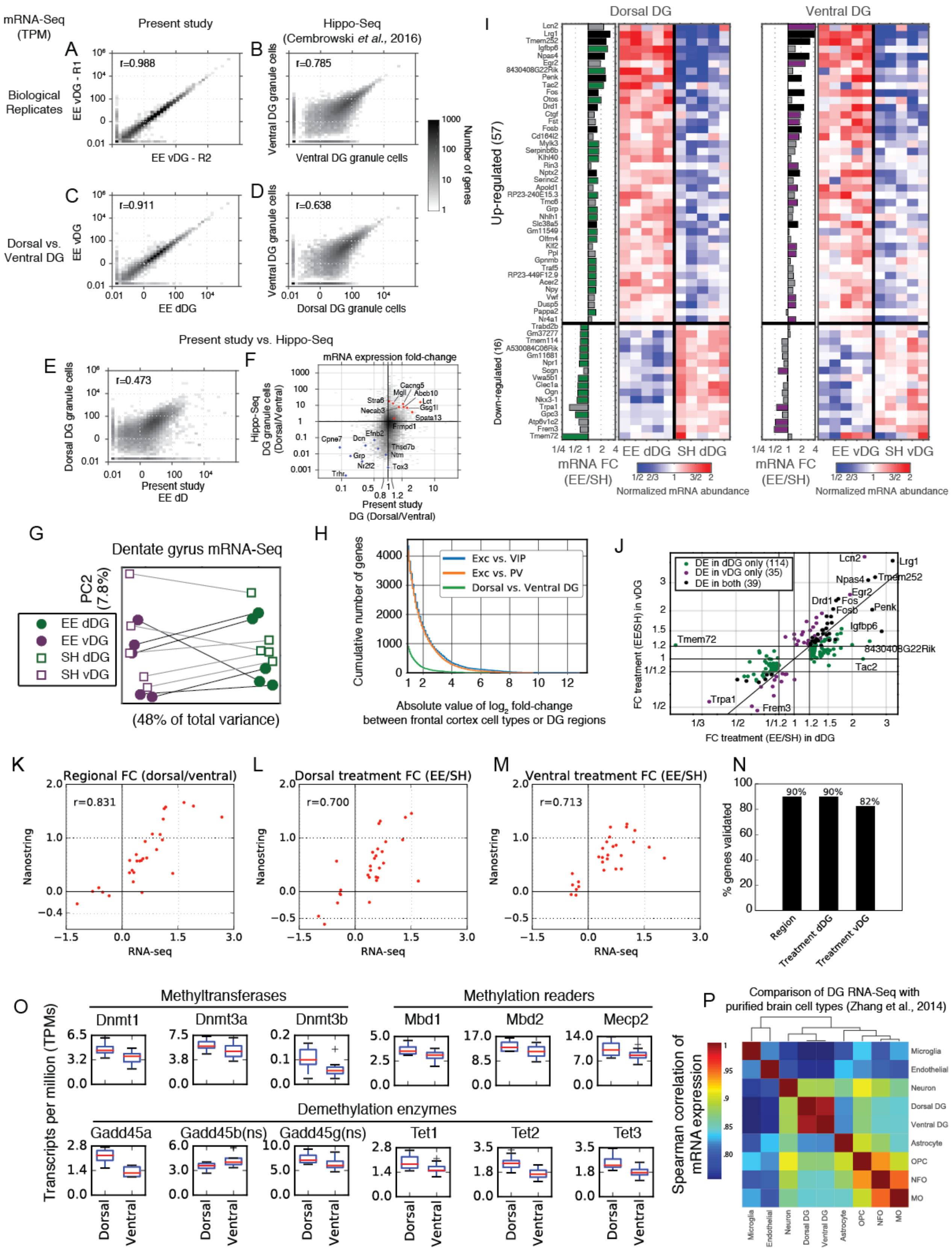

mRNA-Seq (TPM)
Present study
Hippo-Seq (Cembrowski et al., 2016)
A
B
C
D
E
F
G
H
I
J
K
L
M
N
O
P
Biological Replicates
Dorsal vs. Ventral DG
r=0.988
r=0.785
r=0.911
r=0.638
r=0.473
EE vDG - R1
EE vDG - R2
Ventral DG granule cells
EE vDG
EE dDG
Dorsal DG granule cells
Number of genes
Present study vs. Hippo-Seq
Present study EE dD
mRNA expression fold-change
Hippo-Seq DG granule cells (Dorsal/Ventral)
Present study DG (Dorsal/Ventral)
Cacng5
Mgll
Abcb10
Stra6
Lct
Necab3
Gsg1l
Spata13
Fmpd1
Cpne7
Dcn
Efnb2
Thsd7b
Grp
Ntm
Nr2f2
Tox3
Trhr
Dorsal DG
Ventral DG
Up-regulated (57)
Down-regulated (16)
Lcn2
Lrg1
Tmem252
Igfbp6
Npas4
Egr2
8430408G22Rik
Penk
Tac2
Fos
Otos
Drd1
Ctgf
Fst
Fosb
Cd164l2
Mylk3
Serpinb6b
Klhl40
Rin3
Nptx2
Serinc2
Apold1
RP23-240E15.3
Tmc6
Grp
Nhlh1
Slc38a5
Gm11549
Olfm4
Klf2
Ppl
Gpnmb
Traf5
RP23-449F12.9
Acer2
Npy
Vwf
Dusp5
Pappa2
Nr4a1
Trabd2b
Gm37277
Tmem114
A530084C06Rik
Gm11681
Npr1
Scgn
Vwa5b1
Clec1a
Ogn
Nkx3-1
Trpa1
Gpc3
Atp6v1c2
Frem3
Tmem72
mRNA FC (EE/SH)
EE dDG
SH dDG
EE vDG
SH vDG
Normalized mRNA abundance
Dentate gyrus mRNA-Seq
PC2 (7.8%)
(48% of total variance)
EE dDG
EE vDG
SH dDG
SH vDG
Cumulative number of genes
Exc vs. VIP
Exc vs. PV
Dorsal vs. Ventral DG
Absolute value of log2 fold-change between frontal cortex cell types or DG regions
DE in dDG only (114)
DE in vDG only (35)
DE in both (39)
FC treatment (EE/SH) in vDG
FC treatment (EE/SH) in dDG
Regional FC (dorsal/ventral)
Dorsal treatment FC (EE/SH)
Ventral treatment FC (EE/SH)
r=0.831
r=0.700
r=0.713
Nanostring
RNA-seq
% genes validated
90%
90%
82%
Region
Treatment dDG
Treatment vDG
Methyltransferases
Methylation readers
Demethylation enzymes
Dnmt1
Dnmt3a
Dnmt3b
Mbd1
Mbd2
Mecp2
Gadd45a
Gadd45b(ns)
Gadd45g(ns)
Tet1
Tet2
Tet3
Transcripts per million (TPMs)
Dorsal
Ventral
Comparison of DG RNA-Seq with purified brain cell types (Zhang et al., 2014)
Spearman correlation of mRNA expression
Microglia
Endothelial
Neuron
Dorsal DG
Ventral DG
Astrocyte
OPC
NFO
MO

Supplementary Fig. 2: Transcriptome analysis of dorsal and ventral DG in enriched environment. (A,B) Comparison of biological replicates shows the consistency of gene expression estimates (TPM) in ventral DG in the present study (A) and for ventral DG granule cells in Hippo-Seq[2] (B). (C,D) Dorsal vs. ventral expression estimates for the present study (C) and hippo-seq (D). (E) Direct comparison of individual replicates from the present study with hippo-seq granule cells. (F) Comparison of regional differences in expression between the present data set (DG tissue) and purified granule cells from Hippo-Seq (Cembrowski et al. 2016) shows highly consistent differential expression for markers of dorsal and ventral DG granule cells. (G) Effects of EE on gene expression are larger in dDG compared with vDG. (H) Dorsal and ventral DG expression differences are ~4-fold smaller than the differences between distinct cortical cell types. (I) The top DE genes include immediate early genes (*Fos, Egr2, Npas4*). (J) More genes are upregulated (EE>SH) than downregulated (EE<SH). (K-N) Nanostring digital quantification validates RNA-Seq results. (O) Expression of genes associated with (de)methylation and methylation readers in dorsal and ventral DG. All genes are significantly upregulated in dorsal DG over ventral (FDR < .05) except G*add45b*, which is significantly upregulated in ventral DG, and *Gadd45g*, which is not differentially expressed. *Not significant (ns).* (P) Correlation of expression levels between DG tissue and purified brain cell types[16] reveals expression in DG is most strongly correlated with neurons. *Oligodendrocyte progenitor cells (OPC), newly formed oligodendrocytes (NFO), mature oligodendrocytes (MO).*

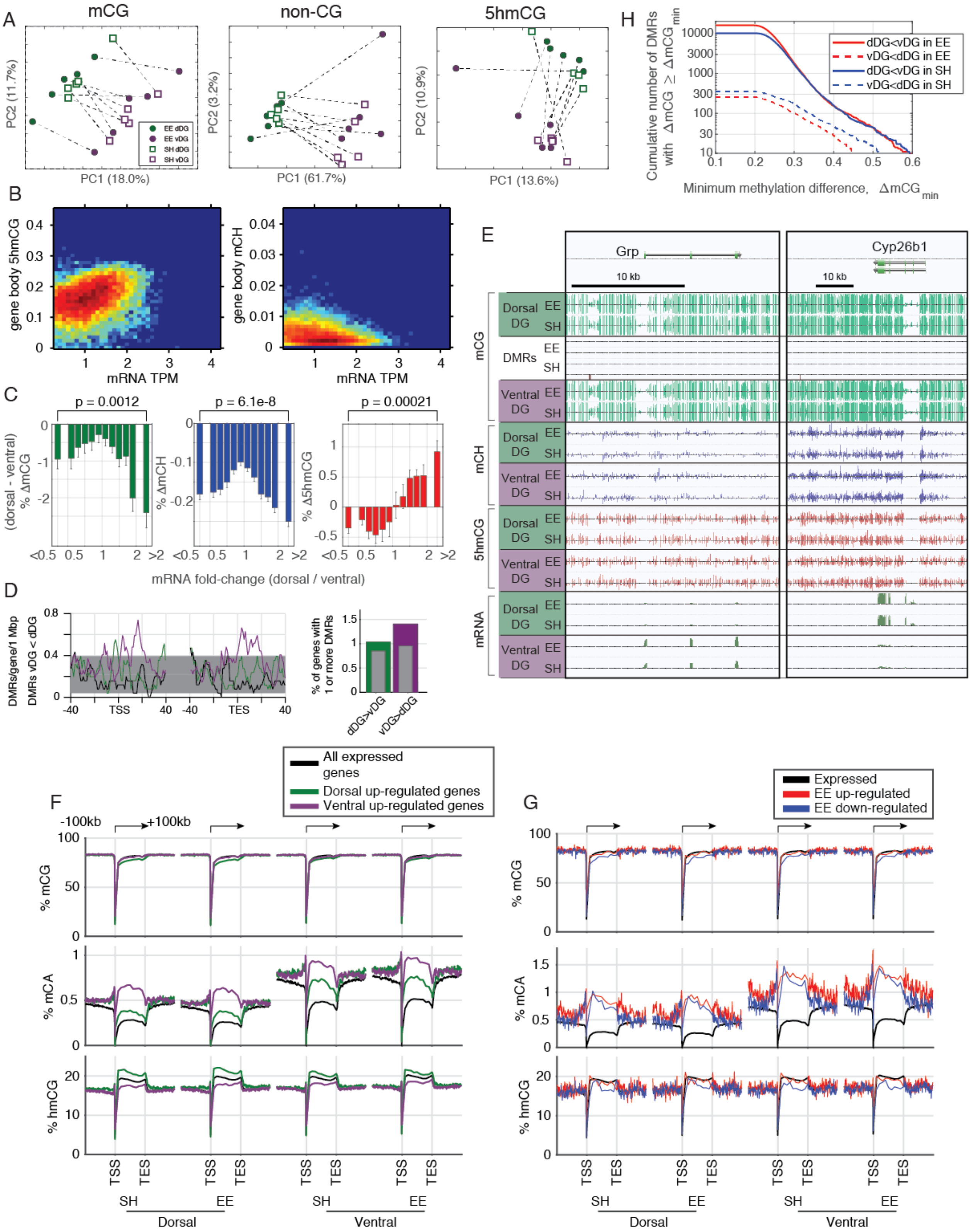
A
mCG
non-CG
5hmCG
PC2 (11.7%)
PC1 (18.0%)
PC2 (3.2%)
PC1 (61.7%)
PC2 (10.9%)
PC1 (13.6%)
EE dDG
EE vDG
SH dDG
SH vDG
H
Cumulative number of DMRs with ΔmCG ≥ ΔmCG min
Minimum methylation difference, ΔmCG min
dDG<vDG in EE
vDG<dDG in EE
dDG<vDG in SH
vDG<dDG in SH
B
gene body 5hmCG
gene body mCH
mRNA TPM
C
p = 0.0012
p = 6.1e-8
p = 0.00021
(dorsal - ventral) % ΔmCG
% ΔmCH
% Δ5hmCG
mRNA fold-change (dorsal / ventral)
D
DMRs/gene/1 Mbp DMRs vDG < dDG
TSS
TES
% of genes with 1 or more DMRs
dDG>vDG
vDG>dDG
E
Grp
Cyp26b1
10 kb
mCG
mCH
5hmCG
mRNA
Dorsal DG
Ventral DG
DMRs
EE
SH
F
All expressed genes
Dorsal up-regulated genes
Ventral up-regulated genes
-100kb
+100kb
% mCG
% mCA
% hmCG
TSS
TES
SH
EE
Dorsal
Ventral
G
Expressed
EE up-regulated
EE down-regulated
% mCG
% mCA
% hmCG

Supplementary Fig. 3. (A) Transcriptome principal components separate dorsal and ventral samples using mCG, mCH or 5hmCG. Dashed lines connect dorsal and ventral DG from the same group of mice. (B) Gene body mCH is associated with transcriptional repression, whereas 5hmCG is positively correlated with expression[21,25]. (C) Genes up-regulated in dorsal vs. ventral DG have lower promoter mCG, gene body mCH, and higher gene body 5hmCG compared with down-regulated genes. (D) Ventral hypo-methylated DMRs are enriched within the gene body of genes up-regulated in ventral DG. (E) Browser shots showing examples of genes up-regulated in ventral (*Grp*) or dorsal (*Cyp26b1*) DG, but which lack DMRs. (F,G) Mean mCG, mCA and hmCG levels within gene bodies ±100 kb are shown for all expressed genes, as well as genes differentially expressed in dorsal or ventral DG. mCA is depleted, and hmCG is enriched, within the gene bodies of dorsal expressed genes (green lines); the opposite pattern prevails within the bodies of ventral expressed genes (purple lines). (H) Number of hypomethylated DMRs in dorsal (solid lines) and ventral (dashed lines) DG as a function of the methylation difference threshold.

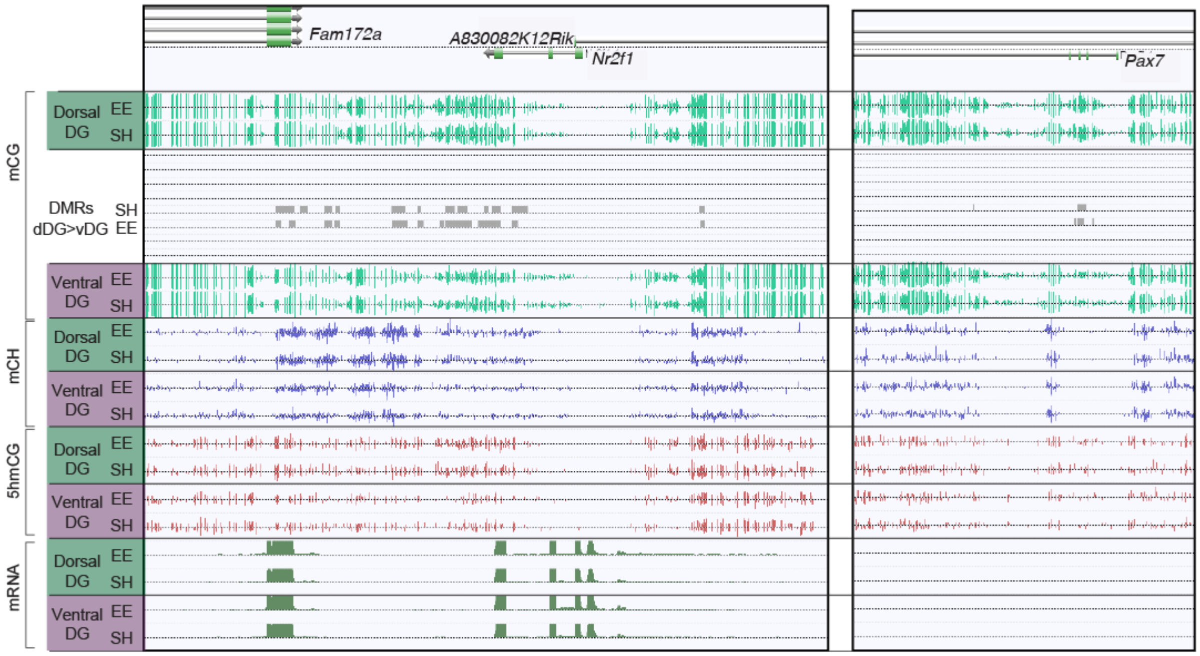

Supplementary Fig 4. Browser views of examples of genes hypomethylated in ventral DG. (Left) *Nr2f1* is differentially expressed in adult ventral DG, consistent with the lower mCG level in the gene body and surrounding region. (Right) *Pax7* is not expressed in the adult DG, but is expressed during development and helps to define dorsal fate[37]. Interestingly, we find that *Pax7* is more methylated in the dorsal DG, which may be a vestigial signature of its early developmental activity in the dorsal DG similar to vestigial DMRs observed in other neuronal cell types[9].

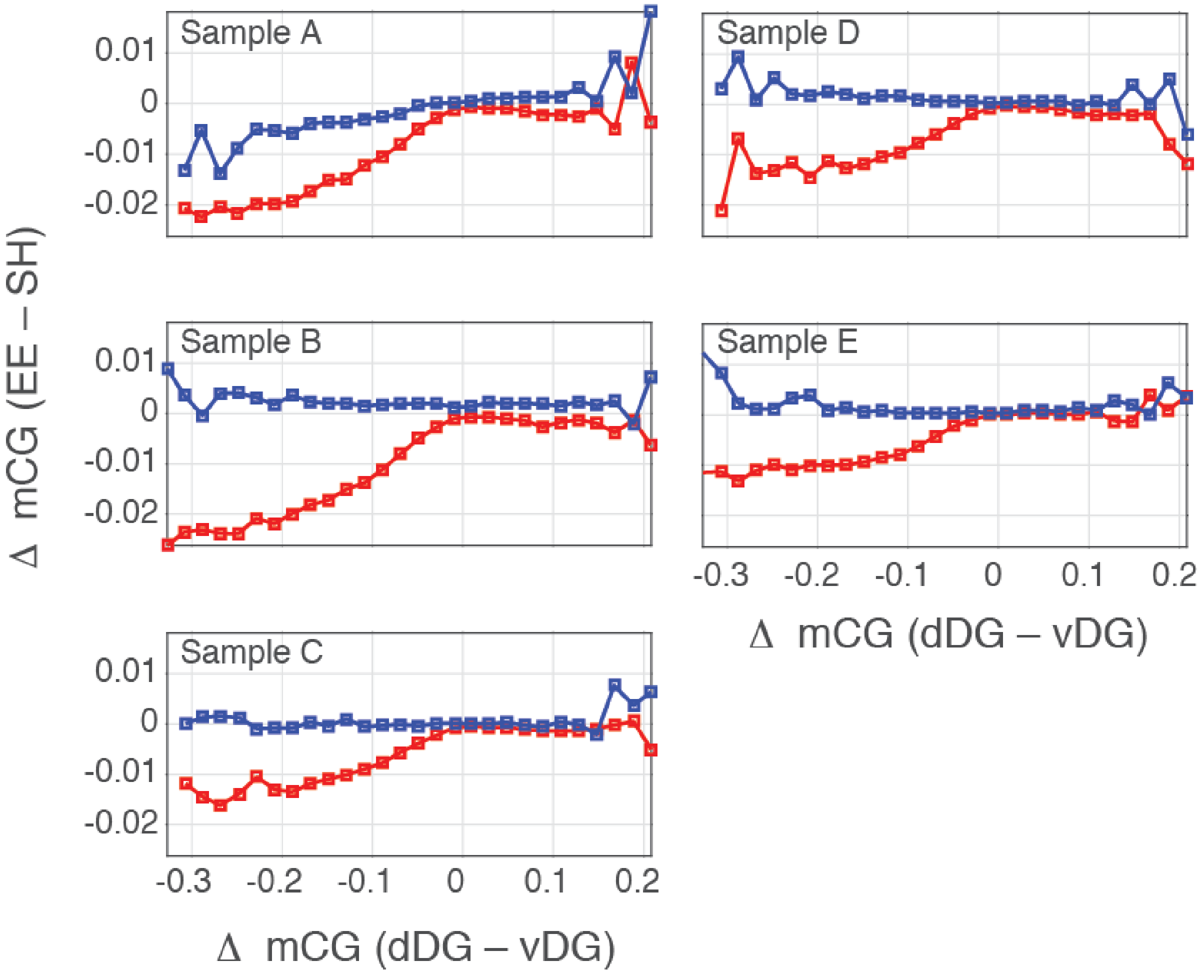

Supplementary Fig. 5. Cross-validated analysis of the correlation of region- and treatment-based effects on DNA methylation. Each panel shows the result of analyzing regional DNA methylation in one of the five biological replicates (samples A-E, x-axis) vs. the treatment-based effects estimated using the remaining four samples. DNA methylation was estimated within 1kb bins across all autosomes; we excluded bins with <10 CG basecalls in any sample, or <100 basecalls after summing all samples. Binned mCG levels were used to compute the dorsal-ventral and EE-SH difference in methylation for each bin. We then grouped all bins according to their level of dorsal-ventral difference in mCG, and computed the median EE-SH difference.
