# Supplementary material for: Environmental enrichment increases transcriptional and epigenetic differentiation between mouse dorsal and ventral dentate gyrus
Source: Nat Commun. 2018 Jan 19;9:298. doi: 10.1038/s41467-017-02748-x (PMC5775256; doi:10.1038/s41467-017-02748-x)
Supplement: Supplementary file 3 — Description of Additional Supplementary Files [file 41467_2017_2748_MOESM3_ESM.pdf]

File Name: Supplementary Data 1
Description: Sample information and sequencing metrics, differential expression analysis, differentially methylated regions (DMRs), and differentially methylated regions overlapping NeuroD1 ChIP-Seq peaks.
